# Understanding the Enzymatic Synthesis of a New Biolubricant: Decane-1,10-diyl bis(2-methylpentanoate)

**DOI:** 10.3390/molecules30010052

**Published:** 2024-12-26

**Authors:** Salvadora Ortega-Requena, Fuensanta Máximo, María Claudia Montiel, María Gómez, María Dolores Murcia, Josefa Bastida

**Affiliations:** Department of Chemical Engineering, Campus de Espinardo, University of Murcia, 30100 Murcia, Spain; dortega@um.es (S.O.-R.); fmaximo@um.es (F.M.); cmontiel@um.es (M.C.M.); maria.gomez@um.es (M.G.); md.murcia@um.es (M.D.M.)

**Keywords:** biocatalysis, branched esters, lipase, esterification, biolubricant, Green Chemistry

## Abstract

The value of branched esters comes from the special properties they have in cold environments, which allow them to remain liquid over a wide range of temperatures. These properties make them useful for application in the cosmetic industry or as lubricant additives. This paper presents the studies carried out to ascertain the operational feasibility of the enzymatic esterification of 2-methylpentanoic acid (MPA) with 1,10-decanediol (DD), with the objective of obtaining a novel molecule: decane-1,10-diyl bis(2-methylpentanoate) (DDBMP). The enzymatic reaction is conducted in a thermostated batch reactor, utilizing the commercially available immobilized lipase Lipozyme^®^ 435 in a solvent-free medium. The reaction conversion is determined by an acid number determination and a gas chromatographic analysis. The most optimal result is achieved at a temperature of 80 °C, a biocatalyst concentration of 2.5% (*w*/*w*), and a non-stoichiometric substrate relation. A preliminary economic study and the calculation of Green Metrics has established that the operation with a 30% molar excess of acid is the best option to obtain a product with 92.6% purity at a lower cost than the other options and in accordance with the 12 Principles of Green Chemistry. The synthetized diester has a viscosity index of 210, indicating that this new molecule can be used as a biolubricant at extreme temperatures.

## 1. Introduction

Biocatalytic processes are considered sustainable processes clearly framed within the well-known Green Chemistry and, therefore, are perceived as a quintessential alternative to traditional production processes. This is mainly due to the high selective capacity of the reaction and the high purity of the final products [1]. The theoretical framework of Green Chemistry is considered a methodology of action that provides science with notions of continuous innovation and research in numerous fields of application [2]. The use of living organisms and enzymes has allowed for the synthesis of compounds on a small scale, but with a high added value for the pharmaceutical industry, whose compounds require regioselective synthesis to obtain very high purities [3,4]. The application of these techniques would allow medium-scale industries, such as food and cosmetics, to mark their products with the natural-compound label [5]. Even on a large scale, it is estimated that a large portion of plastic additives or biolubricants and biodiesel could be easily replaced with biocatalytically synthesized compounds [6].

Lipases constitute one of the most important groups of enzymes in biotechnology, given their versatility in different fields of application and high production yields [7]. Lipases have a high substrate specificity, stereoselectivity, and enantioselectivity and are therefore, frequently used in various biocatalytic reactions [8,9]. Although lipases can be used in their native form, their solubility in aqueous media is a negative factor for correct separation in a reaction medium, which can also be affected by pH, temperature, and the consequent denaturation because they are protein molecules [10]. The high influence of the cost of biocatalysts has promoted the development of enzymatic immobilization techniques that can improve the specificity, activity, and kinetic parameters [11], favoring the recoverability of this biocatalyst and a correct separation of the final products in each type of synthesis in which they are applied. Currently one of the main suppliers of immobilized enzymes, the company Novozymes [12], offers a wide range of immobilized biocatalysts. Lipozyme^®^ 435, a commercial *Candida antarctica* lipase (CalB), previously called Novozym^®^ 435, exhibits a superior performance compared to the other enzymes in both hydrolysis reactions and synthesis in organic solvents and in solvent-free media [13].

One of the most widespread uses of lipase is as a catalyst in the enzymatic synthesis of esters, which are compounds of variable compositions and structures that determine their properties and therefore, their potential applications. Among these, branched esters are employed extensively in both the cosmetic and pharmaceutical industries, as they enhance the dispersion and wetting behavior of the product, particularly when applied to human skin [14]. Lipozyme^®^ is an efficient catalyst for esterification reactions using linear acids and branched alcohols, achieving conversions close to 90% in 1 h [15]. However, this enzyme is less effective when it uses linear alcohols and branched acids as substrates [14]. Studies have linked carboxylic acid structure to lipase activity, affecting its active center [14]. Furthermore, acid pKa values lower than 4.8 have been associated with the irreversible inactivation of an enzyme. This problem is emphasized when the stoichiometric acid–alcohol ratio is 2:1 [16,17].

Nevertheless, due to the widespread use of these esters in the cosmetic and pharmaceutical industries, efforts have been made to produce them by biocatalysis. In particular, attempts have been made to synthesize the esters of 2-ethylhexanoic acid, although these required extreme reaction conditions and yielded results indicating a limited feasibility for industrial use [18,19,20,21]. The research highlights that the length and position of the branched chain in carboxylic acid is critical to the catalytic activity of CalB. As a result, the authors have conducted studies using 2-methylhexanoic acid as a substrate in enzymatic reactions, with promising results that have led to the development of sustainable ester synthesis processes suitable for use as biolubricants [22,23].

**Figure 1:**
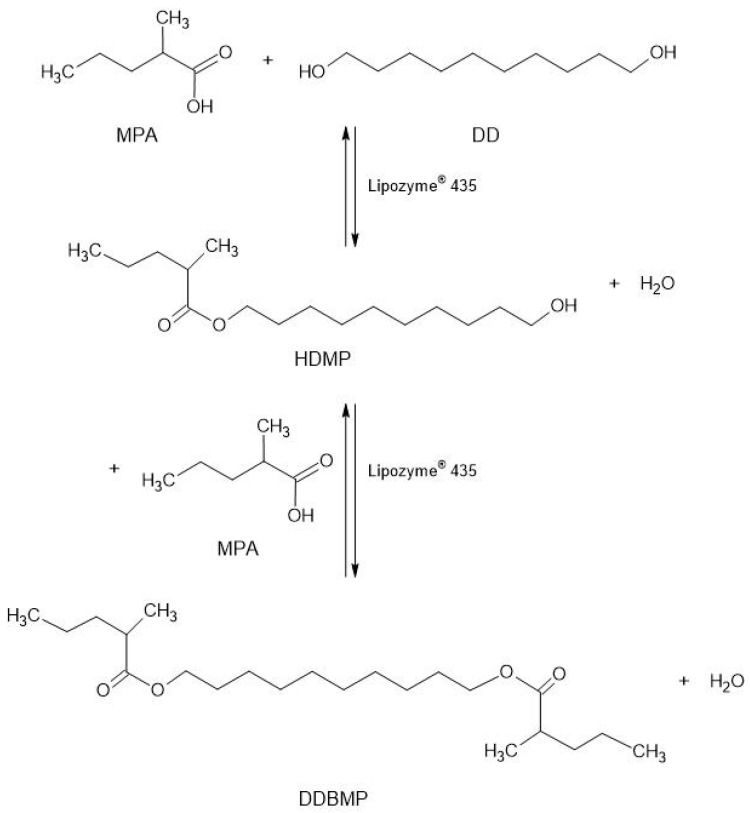
Reaction scheme for the biocatalytic synthesis of a new branched acid diester. MPA: 2-methylpentanoic acid; DD: 1,10-decanediol; HDMP: 10-hydroxydecyl-2-methylpentanoate; DDBMP: decane-1,10-diyl bis(2-methylpentanoate).

In the present paper, as a continuation of the previously mentioned works, the synthesis of the branched diester decane-1,10-diyl bis(2-methylpentanoate) (DDBMP) obtained by a reaction between 2-methylpentanoic acid (MPA) and 1,10-decanediol (DD) has been developed for the first time, without forgetting that the biocatalytic synthesis of this branched acid could trigger several problems such as the following: a lengthening of the reaction time, the vaporization of the acid, a decrease in catalytic activity, or even the inactivation of the enzyme. A scheme of the reaction is shown in Figure 1.

## 2. Results and Discussion

### 2.1. Preliminary Studies

A preliminary study is an essential component for the process of optimizing a biocatalytic synthesis of a new compound. This preliminary investigation determines the suitability of the enzyme, reaction conditions, and potential challenges. The collection of fundamental data allows for more effective optimization strategies, reducing the expenditure of time and resources while enhancing the probability of attaining high yields, selectivity, and efficiency.

**Figure 2:**
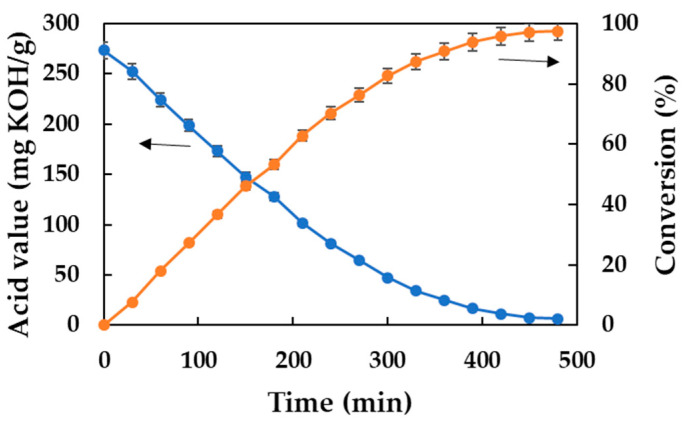
Biocatalytic synthesis of DDBMP. Reaction conditions: 20 g substrates, acid–alcohol molar ratio of 2:1, 70 °C, 350 rpm, and 2.5% (*w*/*w*) Lipozyme^®^ 435 concentration.

Thus, the synthesis of DDBMP was initially carried out at a temperature of 70 °C, with a substrate molar ratio of 2:1, 350 rpm, and a biocatalyst concentration of 2.5% (*w*/*w* with respect to the mass of substrates) to estimate if this reaction could be viable for study and optimization. The results obtained are shown in Figure 2 where the decrease in acid value and the increase in conversion over the reaction time can be observed. These results show that it is indeed possible to carry out the biocatalytic synthesis of this branched diester in a solvent-free reaction medium. This finding is crucial because, to the best of our knowledge, it is the first time that an ester of 2-methylpentanoic acid has been obtained using lipase as a biocatalyst.

### 2.2. Optimization of the Operating Temperature

**Figure 3:**
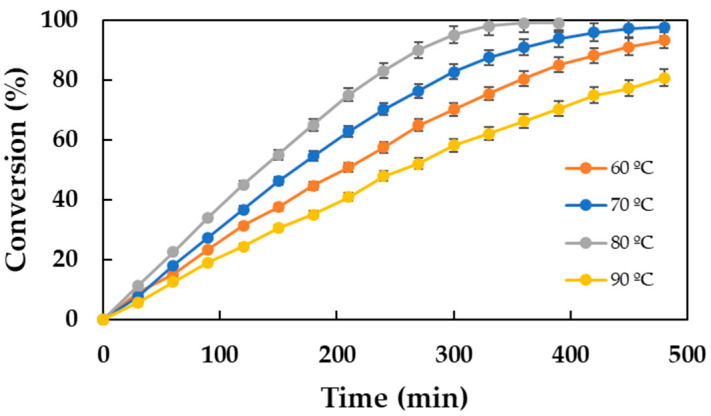
Influence of temperature on the biocatalytic synthesis of DDBMP. Reaction conditions: 20 g substrates, acid–alcohol molar ratio of 2:1, 350 rpm, and 2.5% (*w*/*w*) Lipozyme^®^ 435 concentration.

The kinetics of a chemical reaction are favored by the increase in temperature following the Arrhenius equation. However, in enzyme-catalyzed processes, due to the thermal deactivation of the biocatalyst, it can be observed that, at certain temperatures, the performance may be lower than expected [18]. Therefore, it is of the utmost importance to estimate the optimal operating temperature since it is directly associated with the conversion achieved and the purity of the final product. The results obtained in this study are shown in Figure 3, which represents the evolution of the conversion over time for the tested temperatures between 60 and 90 °C.

It is observed that the reaction rate exhibits a notable increase in concert with the elevated temperatures, attaining its maximal value at 80 °C. At this temperature, a conversion of 99% is achieved after 6 h of reaction time, probably due to a decrease in viscosity. However, for the temperatures of 60 and 70 °C, the conversions are 80.40 and 90.83%, respectively, for an identical time period. However, this phenomenon was not observed when the temperature was increased to 90 °C. At this temperature, the reaction rate exhibited a notable decline, which is contrary to the predictions of the Arrhenius equation. The process is catalyzed by an enzyme (CalB lipase) whose optimal operational temperature range is between 30 °C and 60 °C [15]. At a temperature of 90 °C, the enzyme’s activity is reduced, which results in a notable decrease in the reaction rate and a low conversion rate after a reaction time of 6 h (66.14%). This phenomenon has not been observed in similar reaction systems [23], where CalB activity is maintained at 90 °C. In the present case, the combination of the temperature with the high amounts of MPA could be the cause of the reported loss of activity. Therefore, an optimal temperature of 80 °C is considered.

### 2.3. Influence of the Biocatalyst Concentration

The optimal concentration of the immobilized enzyme in a biocatalytic reaction is a critical determinant of efficiency and cost-effectiveness. An appropriate enzyme concentration is essential to ensure sufficient catalytic activity, avoid overuse, and prevent diffusion limitations. Optimizing the enzyme concentration involves striking a balance between activity, stability, and economic viability, improving the process yields and sustainability.

**Figure 4:**
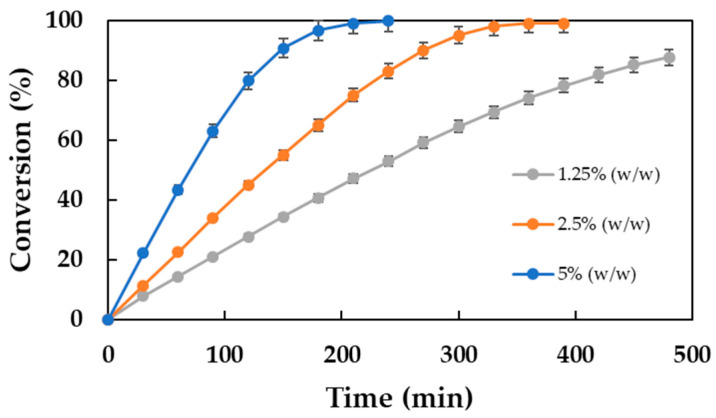
Influence of Lipozyme^®^ 435 concentration on the biocatalytic synthesis of DDBMP. Reaction conditions: 20 g substrates, acid–alcohol molar ratio of 2:1, 350 rpm, and 80 °C.

Accordingly, a series of experimental trials have been undertaken. The concentrations of the enzymes used were 1.25, 2.5, and 5% (*w*/*w*) with respect to the mass of the substrates, at a constant temperature of 80 °C. The remaining operational parameters were maintained at the same levels as in the preceding experiments: stirring occurred at 350 rpm and a molar ratio of 2:1 (acid–alcohol) was used. The results obtained are shown graphically in Figure 4, where the conversions achieved over time for the different enzyme concentrations have been represented. As can be seen, both the reaction rate and the final conversion increase when the biocatalyst concentration increases. With a biocatalyst concentration of 1.25%, a conversion of 87.66% is achieved after 8 h of reaction time. Increasing the concentration to 2.5% results in 99% conversion in only 6 h. This reaction time can be further reduced to 4 h by increasing the immobilized enzyme concentration to 5%. However, the use of such a high biocatalyst concentration has operational challenges, such as poor mass transfer due to the high solids content in the reaction medium and high operational costs due to the elevated price of the biocatalyst. Therefore, a 2.5% concentration is assumed to be an adequate level for subsequent experiments.

### 2.4. Biocatalyst Operational Stability

**Figure 5:**
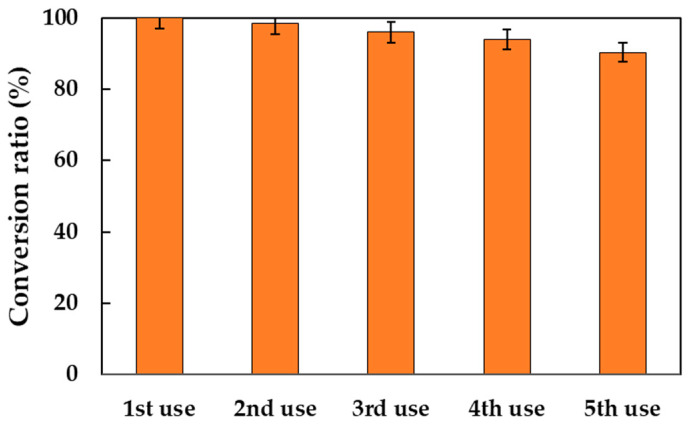
Reusability test of Lipozyme^®^ 435 for DDBMP synthesis. Reaction conditions: 20 g substrates, acid–alcohol molar ratio of 2:1, 80 °C, 350 rpm, and 2.5% (*w*/*w*) biocatalyst concentration.

It is crucial to assess the stability of immobilized enzymes in industrial processes to ensure cost-effective, sustainable, and efficient reactions. The operational stability of Lipozyme^®^ 435 is determined after several consecutive cycles of reaction, separation, and reuse. Figure 5 illustrates the progression of the conversion rate for five reaction cycles. As can be observed, the biocatalyst retains 90% of its initial activity, which indicates that the reuse of the immobilized enzyme markedly enhances productivity, resulting in a notable increase from 45.12 kg DDBMP/kg of biocatalyst to 116.72 kg DDBMP/kg of biocatalyst after the five uses.

There results differ slightly from those obtained previously [23], where the same immobilized lipasa maintains 96% of its activity after five reaction cycles when used for the 2-octyl-1-dodecanoyl-2-methylhexanoate ester synthesis. As discussed in the Introduction Section, this difference may be due to two different reasons. On the one hand, in the present study the ester is produced from an acid and a diol, so the stoichiometric molar ratio of substrates is 2:1, while in the process referenced in the literature this ratio is 1:1; therefore, the acid concentration in the present reaction is higher. Therefore, the deactivating effect of the acid medium is increased. On the other hand, it is widely understood that the pKa of the acid involved in biocatalytic synthesis plays a very important role in this reaction. Thus, it has been asserted that acids with a pKa value lower than 4.8 can cause the irreversible inactivation of *Candida antarctica* lipase B [16,17]. MPA has a pKa of 4.782 [24], which, although close to 4.8, is low enough to exert such an inactivating effect.

### 2.5. Monitoring the Reaction Through a Gas Chromatography Analysis

**Figure 6:**
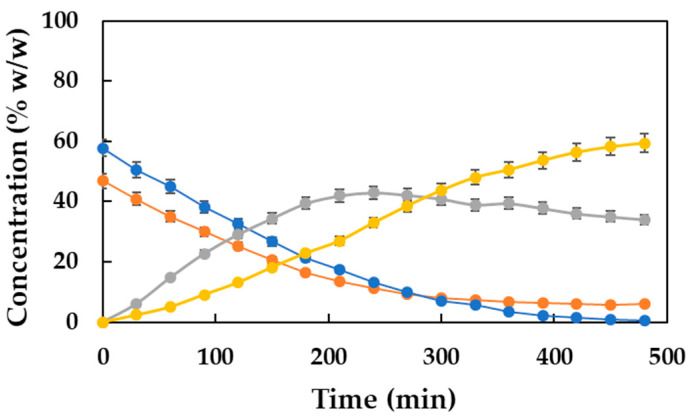
Evolution of the concentration (% *w*/*w*) of both substrates (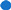 MPA and 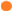 DD), monoester (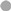 HDMP), and diester (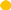 DDBMP) with time. Reaction conditions: 20 g of substrates, acid–alcohol molar ratio of 2:1, 80 °C, 350 rpm, and 2.5% (*w*/*w*) biocatalyst concentration.

A reaction was carried out under the optimal conditions previously established (80 °C temperature, 2.5% (*w*/*w*) biocatalyst concentration, 350 rpm stirring, and a stoichiometric acid–alcohol molar ratio of 2:1), and an analysis of the reactor samples was performed via gas chromatography. In this way, the evolution of the different substances present in the reaction mixture can be monitored and the purity of the final product obtained. The progressions of the concentration of the substrates (MPA and DD), reaction intermediate (HDMP), and product (DDBMP) with the reaction time are shown in Figure 6. As can be observed, the concentrations of both substrates decrease with time while that of the intermediate HDMP increases initially, up to 6 h, and then decreases slightly. On the other hand, the curve corresponding to DDBMP shows a progressive increase of the product concentration in the reaction medium. After 7.5 h, however, the acid concentration is virtually zero, while the reaction medium still contains significant amounts of both unreacted DD and intermediate HDMP that have not been converted to DDBMP. As a consequence of this fact, the final concentration of the diester is quite low (59.43% *w*/*w*).

Obviously, the disappearance of the acid cannot be attributed exclusively to its consumption as a substrate for the reaction since a considerable amount of the intermediate and the other substrate still remain in the medium. Therefore, this fact can be attributed to a loss of the compound by evaporation since the reaction temperature is relatively high (80 °C). This phenomenon has been observed in other enzymatic reactions, although it always referred to alcohol [14,17]. To test this hypothesis and solve the problem caused, the available literature resorts to carrying out the reactions with initial substrate molar ratios slightly higher than the stoichiometric one, thus compensating for the aforementioned loss [14,17].

### 2.6. Study of the Biocatalytic Synthesis of DDBMP with an Excess of Acid

**Figure 7:**
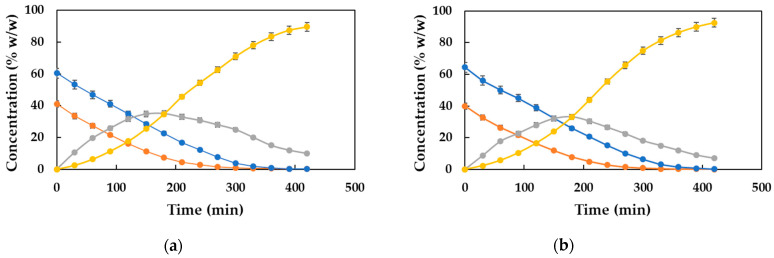
Evolution of the concentration (% *w*/*w*) of both substrates (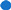 MPA and 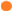 DD), monoester (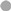 HDMP), and diester (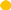 DDBMP) with time. Reaction conditions: 20 g of substrates, 80 °C, 350 rpm, and 2.5% (*w*/*w*) biocatalyst concentration. (**a**) Acid–alcohol molar ratio of 2.4:1 and (**b**) acid–alcohol molar ratio of 2.6:1.

In light of the above and to improve the productivity of the process in terms of obtaining the diester, a feasible alternative is carrying out the esterification reaction using an excess of acid. In the present study, the molar excesses of acid used have been 20 and 30%, which correspond to the acid–alcohol ratios of 2.4 and 2.6 to 1, respectively. The reaction was carried under the optimal conditions for temperature (80 °C), biocatalyst concentration (2.5% (*w*/*w*)), and stirring speed (350 rpm). The monitoring of both reactions were carried out by analyzing the reaction samples by gas chromatography. The results obtained are shown in Figure 7 (a and b), which shows the evolution of the concentration of the different species present in the reaction medium over time. It is observed that the substrates are completely consumed after 5–6 h of reaction time. On the other hand, the intermediate monoester HDMP reaches its maximum value for a time of around 3.5 h, and begins to slowly decrease to a value close to zero after 7 h of reaction time, agreeing with an increase in the concentration of the diester DDBMP. In these circumstances, the concentrations of the final product are very high: 89.6% (*w*/*w*) for the synthesis carried out with 20% molar excess acid and 92.6% (*w*/*w*) when using 30% excess acid.

**Figure 8:**
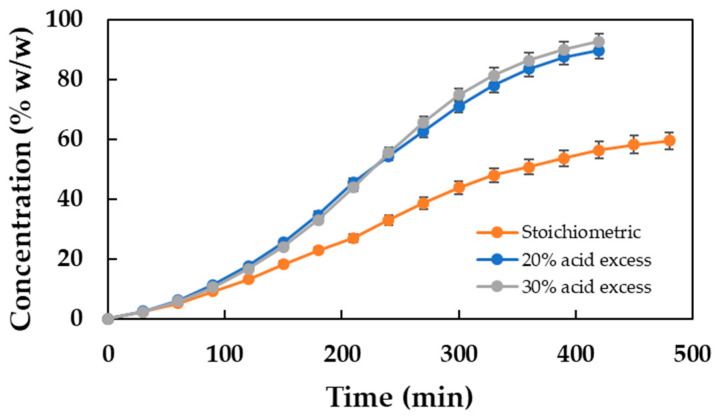
Evolution with reaction time of the concentration (% *w*/*w*) of DDBMP. Reaction conditions: 20 g of substrates, 80 °C, 350 rpm, and 2.5% (*w*/*w*) biocatalyst concentration.

In view of what is shown in Figure 7, it seems the results obtained represent a clear improvement when compared to those obtained when the reaction was carried out with stoichiometric amounts of the substrates (illustrated in Figure 6). For a more accurate comparison, the time evolutions of the DDBMP concentrations in the three reaction conditions have been plotted together in Figure 8, enabling a more comprehensive evaluation.

The figure clearly shows that the final concentration reached is much higher for the two experiments carried out with excess acid, being slightly better for the 30% excess. The productivity of the process is improved from 0.063 kg DDBMP/L h (stoichiometric) to 0.105 kg DDBMP/L h (20% acid excess) and 0.112 kg DDBMP/L h (30% acid excess). Moreover, the purity of the final product increases from 59.5% to 86.9% and 92.6%, respectively.

In light of these results, it may be recommended that the biocatalytic synthesis of DDBMP be performed with 30% excess acid. It is, however, important to note that the aforementioned operating conditions would result in increased production costs and a reduction in the sustainability of the process. It is therefore evident that the ultimate decision should be based on a comprehensive analysis of the economic and environmental impacts.

### 2.7. Setting Optimal Reaction Conditions Based on Preliminary Economic Studies and Green Metrics

A preliminary economic study has been conducted in order to identify the optimal initial concentration of MPA. The direct manufacturing costs for 1 kg of DDBMP have been calculated as the sum of the operating costs and those associated with the substrates and biocatalysts utilized in the production process. Table 1 presents the prices of these compounds, acquired in bulk, the cost of Lipozyme^®^ 435, and the energy expenses (thermostatic bath and vertical stirrer) per time unit. The final three columns present the costs associated with each item for the different acid concentrations tested: stoichiometric, 20% excess, and 30% excess. Additionally, the total manufacturing costs for 1 kg of the diester DDBMP are provided.

It is clear that the cost of producing 1 kg of DDBM is lower for all the items considered when the biocatalytic synthesis is carried out with 30% excess acid, due to the higher productivity of this process. However, given that the difference between the predicted cost for 20% excess and 30% excess is only 4.5%, the environmental impact of using excess acid also needs to be considered before a final decision is made [25].

To accurately determine the environmental viability of a process, it is necessary to establish numerical measures and not rely exclusively on qualitative considerations. This quantitative information is obtained by determining sustainability indicators, called “Green Metrics”. The most commonly used are shown in Table 2 for the two selected processes: those with 20% and 30% excess acid [1].

For both processes, the same value for Atom Economy (AE) is obtained since its calculation is based on the stoichiometry of the reaction [26]. This value is higher than 90%, indicating that most of the substrates have been incorporated into the desired product with a minimal production of by-products, and the only waste is the water released in the reaction. The environmental factor (E-factor) is a quantification of all the waste in a process, and its ideal value must obviously be close to zero. The existing literature indicates that two distinct types of E-Factors should be calculated: the simple E-Factor and the complete E-Factor, which incorporates water as a waste component [27,28]. The E-factor values calculated for both processes (0.61 for 20% excess and 0.62 for 30% excess) are sufficiently low to ensure the sustainability of the aforementioned processes. The observed difference from the previously published values for similar procedures [23] can be attributed to the loss of part of the MPA by evaporation. In addition to this fact, in the case of cEF, the stoichiometry of the reaction itself also plays a role, in which two moles of the water are released for each mole of DDBMP produced.

The CME is defined as the percentage of carbon in the reagents that will remain in the final product, and its value should be close to 100% [4,29]. As can be seen in Table 2, the CME values obtained in the two processes studied are significantly lower than desired (60% approximately). The carbon loss due to the evaporation of the MPA mentioned above accounts for this discrepancy. In the same way, this fact determines that the PMI indicator presents a value slightly higher than unity, which is the ideal figure [30]. However, both values are not sufficiently unfavorable to conclude that the biocatalytic syntheses studied cannot be considered sustainable and environmentally friendly.

Overall, the calculated Green Metrics indicate that the biocatalytic synthesis of the new DDBMP diester is a sustainable process, whether it is carried out with 20% or 30% excess acid. Most importantly, the EF values are lower than those reported in the literature for bulk chemical production methods [31]. However, the similarity of the metrics obtained for the two acid excesses does not make it possible to discriminate between them and thus determine the optimum process conditions, which was the aim of this study. Therefore, and in view of the results of the economic study, it can be affirmed that the use of a 30% molar excess of MPA makes it possible to obtain a DDBMP with a higher purity and at a lower cost than when carried out with a 20% excess of acid, with both processes being environmentally friendly.

### 2.8. Characterisation of Decane-1,10-diyl bis(2-methylpentanoate) as a Biolubricant

The viscosity index (VI) is perhaps the most useful indicator of the quality and properties of a lubricant. This indicative measure is employed to quantify the extent of a change in the viscosity of lubricants in response to alterations in temperature. A higher viscosity index is indicative of a reduction in the magnitude of the change in viscosity that will occur as a consequence of a variation in the temperature [32]. The viscosity index was calculated following ASTM D2270 [33], which requires values for density, dynamic viscosity, and kinematic viscosity at 40 °C and 100 °C. The ASTM standard offers distinct approaches based on whether the viscosity index is below or above 100. Additionally, it is important to note that this method is only valid for substances with a kinematic viscosity above 2 mm^2^/s at 100 °C. Table 3 illustrates the findings for the synthesized DDBMP (92.6% purity), produced via a reaction conducted at 80 °C, with a 2.5% (*w*/*w*) biocatalyst concentration, 350 rpm, and a 30% molar excess of acid.

Using these values and applying the procedure described in the standard [33], the VI of the new branched DDBMP ester was determined to be 210.01. Conventional mineral oil has a viscosity index between 95 and 100. The VI of a highly refined (hydrotreated) mineral oil can be as high as 120. A hydrocracked base oil will have a VI greater than 120, while synthetics may have a higher VI [34]. Most of the biolubricants reported in the literature for the esters obtained by biocatalysis had VIs ranging between 120 and 208 [23,35,36,37,38,39,40,41,42]. Accordingly, this diester is an appropriate choice for use as a biolubricant due to its capacity to maintain a relatively constant viscosity value across a broad temperature range, thereby ensuring consistent performance and protection against wear, even under extreme conditions. Moreover, biolubricants that possess elevated viscosity indexes serve to diminish friction and enhance the overall efficiency of hydraulic systems. Such improvements may potentially result in reduced energy expenditures, particularly with regard to heavy machinery wherein hydraulic systems constitute a principal contributor to power consumption.

## 3. Materials and Methods

### 3.1. Materials

Lipozyme^®^ 435, and CalB lipase immobilized on a hydrophobic acrylic resin with a specified activity of 10,000 PLU/g, were kindly donated by Novozymes Spain S.A. (Madrid, Spain).The 2-methylpentanoic acid (≥98%) and 1,10-decanediol (98%) were obtained from Sigma-Aldrich in St. Louis, MO, USA. All the other reagents used were of analytical reagent grade.

### 3.2. Biocatalytic Synthesis

DDBMP was produced in a 50 mL open air jacketed batch reactor. The reactions were performed without a solvent. A mixture containing 2-methylpentanoic acid (MPA) and 1,10-decanediol (DD) in a 2:1 molar ratio (acid–alcohol) with a total mass of 20 g was added to the reactor. The mixture was stirred using an overhead paddle stirrer (Ika Nanostar 7.5 digital, Barcelona, Spain) at a speed of 350 rpm. Different amounts of the immobilized enzyme (0.25, 0.5, and 1 g, corresponding to 1.25, 2.5, and 5% *w*/*w* relative to the substrates) were tested at temperatures between 60 °C and 90 °C. The reaction progress was followed by determining the acid values of the samples collected from the reactor at various times.

The acid value (AV) is defined as the amount of potassium hydroxide, in milligrams, needed to neutralize the free acids in 1 g of a sample [43]. The conversion can be calculated using the following formula:

$$Conversion\left(\%\right)=\frac{{AV}_{0}-{AV}_{t}}{{AV}_{0}}\times 100$$

where AV_0_ represents the initial acid value at the beginning of the reaction, and AV_t_ is the acid value at a specific time during the process. The results reported in this study are an average of three separate measurements, with errors consistently less than 5%. The graphs include the error bars (±standard deviation).

### 3.3. Biocatalyst Recovery and Reuse

In order to evaluate the potential for reuse of the biocatalysts, a series of ester synthesis assays were conducted under the optimal reaction conditions and with the substrates with a molar ratio of 2:1. Following its utilization, the immobilized enzyme was recovered by simply separating the reaction liquid with a Pasteur pipette and storing it at a low temperature overnight, without any further washing or purification, until the subsequent experiment, in which new reagents were employed.

### 3.4. GC Analysis

The concentrations of both substrates (MPA and DD), the reaction intermediate monoester (HDMP), and the desired diester product (DDBMP) were determined by injecting 1 µL of the diluted sample into an Agilent 7820A (Agilent Technologies Spain SL, Madrid, Spain), gas chromatograph equipped with a flame ionization detector and an Agilent Technologies HP-5 capillary column (30 m × 0.32 mm × 0.25 µm). The injector temperature was set at 250 °C, with a 2:1 split ratio and 1 mL/min nitrogen as the carrier gas. The initial oven temperature was 80 °C, held for one minute and increased to 120 °C at a rate of 75 °C/min, which was also held for one minute. The oven temperature was then increased to 290 °C at a ramp rate of 20 °C/min, held for 3.5 min. The detector temperature was 300 °C. The composition of the samples was determined using methyl myristate as the internal standard (IS). The concentrations were calculated from the corresponding calibration curves and expressed as % (*w*/*w*). In order to obtain the calibration curve corresponding to the reaction product DDBMP, a synthesis reaction was carried out under the conditions given in Section 3.2 with 30% excess acid and standing for 24 h. The concentration of the reaction intermediate HDMP was calculated by the difference with respect to the total mass injected.

**Figure 9:**
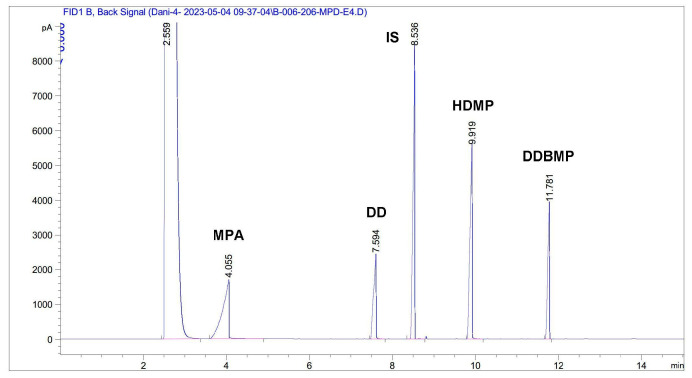
GC analysis of an intermediate sample of the esterification process between 2-methylpentanoic acid (MPA) and 1,10-decanediol (DD). HDMP: 10-hydroxydecyl-2-methylpentanoate; DDBMP: decane-1,10-diyl bis(2-methylpentanoate).

Figure 9 shows a standard chromatogram showing the retention times of the different compounds present in the reaction medium. No peak overlap was observed.

### 3.5. Energy Consumption

To ascertain the energy costs, the present intensity was gauged in real time with an ammeter clamp. It was assumed that the standard voltage at the terminals of the equipment was 220 V. The amount of energy required to maintain the temperature of the reactors by means of thermostatic baths was determined. In order to achieve the requisite temperatures and ensure the uniformity of the reaction mixture, the equipment must first be operated continuously for a period of 10 min. Following this interval, the thermostat transitions into maintenance mode, which reduces energy consumption. Furthermore, the power requirements for the mixers remain constant and significantly lower over time. The estimated energy price was 0.2336 EUR/kW h, representing the average value for July 2024 in Spain.

### 3.6. Characterization of DDBMP

The density and viscosity of DDBMP were determined using a DMA 5000 M density meter/LOVIS 2000 M rolling-ball viscometer from Anton Paar in Madrid, Spain. The measurements were conducted at two distinct temperatures, specifically 40 °C and 100 °C. The viscosity index (VI) was calculated in accordance with the ASTM D2270 standard [33].

## 4. Conclusions

This study demonstrates that the biocatalytic synthesis of the new diester decane-1,10-diyl bis(2-methylpentanoate), obtained from a diol (1,10-decanediol) and a branched acid (2-methylpentanoic acid), is indeed feasible. This represents the inaugural instance of CalB being documented as capable of catalyzing a reaction involving this branched acid. The enzymatic esterification process was conducted at 80 °C with a biocatalyst concentration of 2.5% (*w*/*w*) and a stoichiometric substrate molar ratio of 2:1 acid–alcohol. The resulting final product exhibited a purity of only 59%, which can be attributed to the loss of acid due to vaporization. The process is markedly enhanced by employing a 20% or 30% molar excess of acid. Under these conditions, a final product with 89.59% or 92.6% purity, respectively, can be obtained after 7 h of reaction time. The economic study and Green Metrics have permitted a differentiation between the two acid concentrations, with the operation utilizing a 30% molar excess of 2-methylpentanoic acid being the most advantageous.

The double branching structure and high molecular weight of the newly synthetized ester contribute to the strong lubricating properties, as evidenced by its high viscosity index of 210. Furthermore, it is a saturated compound, offering excellent resistance to oxidation. Both properties render the substance an excellent candidate for use as a biolubricant.

This study paves the way for a new avenue of research, offering the potential to create novel branched molecules through enzymatic esterification between a branched acid and an alcohol. These compounds have the potential to serve as highly effective lubricants and are also biodegradable. Moreover, their synthesis aligns with the 12 Principles of Green Chemistry, making them an environmentally friendly option.

## Figures and Tables

**Table 1:** Substrates, biocatalysts, and energy prices. DDBMP total direct production costs using stoichiometric acid concentration, 20% acid excess, and 30% acid excess.

	Cost	Cost (EUR/kg DDBMP)		
		Stoichiometric	20% Excess	30% Excess
2-Methylpentanoic acid ^1^	30 EUR/kg	11.77	7.38	7.04
1,10-Decanediol ^2^	1 EUR/kg	0.29	0.14	0.14
Lipozyme^®^ 435 ^3^	1600 EUR/kg	27.42	15.52	14.81
Thermostatic bath				
Initial	6.8 10^−3^ EUR/min	11.65	6.60	6.30
Maintenance	8 10^−4^ EUR/min	57.58	32.59	31.11
Overhead stirrer	10^−4^ EUR/min	7.37	4.17	3.98
Total direct cost		116.08	66.40	63.38

^1^ https://tzrunlong.en.made-in-china.com/product/oEDYLgwMbrRZ/China-Fema-3437-3-Methylpentanoic-Acid-CAS-105-43-1.html?pv_id=1ias6e3n4a43&faw_id=1ias6e4eke25 (accessed on 18 November 2024). ^2^ https://leaderbiogroup.lookchem.com/products/CasNo-112-47-0-China-Biggest-Factory-manufacturer-supply-1-10-Decanediol-27312606.html (accessed on 18 November 2024). ^3^ Gift. Personal communication.

**Table 2:** Green Metrics for the biocatalytic synthesis of DDBMP with 20% and 30% acid excess.

Green Metric	Equation	20% Excess	30% Excess
Atom economy (AE)	$AE=\frac{molecular\;weight\;of\;desired\;product}{\Sigma molecular\;weight\;of\;all\;substrates}\cdot 100$	91.14	91.14
E factor (EF)	$EF=\frac{kg\;of\;waste}{kg\;of\;desired\;product}$	0.61	0.62
Complete E factor (cEF)	$cEF=\frac{kg\;of\;waste\;(including\;water)}{kg\;of\;desired\;product}$	0.71	0.72
Carbon mass efficiency (CME)	$CME=\frac{kg\;of\;carbonated\;desired\;product}{kg\;of\;carbonated\;reactants}\cdot 100$	60.08	59.54
Process mass intensity (PMI)	$PMI=\frac{kg\;of\;all\;materials}{kg\;of\;desired\;product}$	1.66	1.68

**Table 3:** Density, dynamic viscosity, and kinematic viscosity values at 40 °C and 100 °C for DDBMP 92.6% purity.

**Temperature (°C)**	**Density (g/cm^3^)**	**Dynamic Viscosity** **(mPa s)**	**Kinematic Viscosity** **(mm^2^/s)**
40	0.903	6.912	7.654
100	0.861	2.256	2.620

## Data Availability

The data presented in this study are available upon request to the corresponding author.
